# Front-Line Maintenance Therapy in Advanced Ovarian Cancer—Current Advances and Perspectives

**DOI:** 10.3390/cancers12092414

**Published:** 2020-08-25

**Authors:** Thibaut Reverdy, Christophe Sajous, Julien Péron, Olivier Glehen, Naoual Bakrin, Witold Gertych, Jonathan Lopez, Benoit You, Gilles Freyer

**Affiliations:** 1 Oncology Department, Hôpital Lyon Sud, Institut de Cancérologie des Hospices Civils de Lyon (IC-HCL) and Université de Lyon, 69310 Lyon, France; christophe.sajous@chu-lyon.fr (C.S.); julien.peron@chu-lyon.fr (J.P.); benoit.you@chu-lyon.fr (B.Y.); gilles.freyer@univ-lyon1.fr (G.F.); 2 Departement of Surgical Oncology, Centre Hospitalier Lyon-Sud, 69310 Lyon, France; olivier.glehen@chu-lyon.fr (O.G.); naoual.bakrin@chu-lyon.fr (N.B.); 3 EA CICLY, Lyon 1 University, 69310 Lyon, France; witold.gertych@chu-lyon.fr; 4 Department of Gynecological Surgery, Centre Hospitalier Lyon-Sud, 69310 Lyon, France; 5 Biochemistry and Molecular Biology Department, Hopital Lyon Sud, Hospices Civils de Lyon, 69310 Lyon, France; jonathan.lopez@chu-lyon.fr

**Keywords:** ovarian cancer, front-line maintenance therapy, PARP inhibitors, anti-angiogenics, immunotherapy, *BRCA 1/2*, homologous recombination deficiency

## Abstract

Ovarian tumor is the gynecological cancer associated with the highest mortality. Most diseases are diagnosed at an advanced stage, which impairs the chances of prolonged complete remission. The standard front-line treatment of advanced stages combines surgery in an expert center with platinum-based chemotherapy. Most patients experience a relapse in the years following the initial treatment. During the last decade, anti-angiogenic agents used in the maintenance setting improved progression free survival (PFS) over chemotherapy alone. More recently, PARP inhibitors demonstrated substantial efficacy, mainly in patients with germinal or somatic *BRCA* mutations or other homologous recombination deficiencies (HRD), all involved in double strand DNA Damage Repair (DDR). Other therapeutic paradigms are currently being explored, including combinations of immune-checkpoints inhibitors, chemotherapy, bevacizumab and PARP inhibitors. In addition to these clinical advances, molecular characterization of the tumors and their correlations with drugs efficacy are needed to better understand which patient will benefit the most from the various treatments available to date.

## 1. Introduction

Ovarian cancer is the deadliest gynecological cancer in the world with 295,414 new cases in 2018 and 184,799 deaths according to GLOBOCAN [1]. More than 75% of patients are diagnosed at an advanced stage of the disease (defined by International Federation of Gynecology and Obstetrics (FIGO, https://www.figo.org) stage IIB to IV) and their five-year survival rate is around 25%. The best outcomes in advanced epithelial ovarian cancer (AEOC) have been observed in patients who were treated with front-line complete surgical resection of the intra-abdominal tumor followed by adjuvant platinum-taxane chemotherapy. The aim of the debulking surgery is complete cytoreduction of all visible macroscopic disease, since this has been shown to be associated with a significant increase in overall survival (OS) and progression free survival (PFS) [2]. This procedure must be performed by well-trained gynecological oncology surgeons [3,4]. Regarding chemotherapy, platinum containing regimens have been the standard of care for decades. Paclitaxel combined with platinum has demonstrated an increase in overall survival over single-agent platinum therapy [5]. Hence carboplatin and paclitaxel doublet administered weekly or every three weeks for six to nine cycles has been the gold-standard for decades [6,7].

After surgery and chemotherapy (frontline followed by adjuvant or neo-adjuvant and interval debulking surgery, IDS), 70% of patients with AEOC will achieve complete response (CR) but more than 90% of them will eventually relapse. Most of the relapses will not be curable and treatment efficacy will decrease with time. Various complications impairing quality of life will occur, such as bowel obstruction, ascites and pleural effusion. Death will occur after a median of 30–35 months in most cases but the prognosis may be even poorer in patients with platinum-resistant diseases.

In that context, the concept of maintenance treatment was developed as a way of keeping the disease free of progression as long as possible after first line medical-surgical treatment in patients with tumors under control (according to the Response Evaluation Criteria in Solid Tumours (RECIST)) at the end of chemotherapy, regardless of the stage of the disease and the amount of residual disease. Before the bevacizumab and PARP inhibitors era, various agents, such as the anti-CA 125 antibody abagovomab [8] or chemotherapy agents (paclitaxel, topotecan [9,10,11]) failed to demonstrate any efficacy.

## 2. Targeting Tumor Endothelium: The Anti-Angiogenic Agents

Vascular endothelial growth factor (VEGF) promotes angiogenesis, along with cancer cells spreading through the vascular system [12]. In two randomized phase III trials, bevacizumab, a monoclonal humanized antibody targeting the circulating VEGF was evaluated in the front-line setting in women with advanced ovarian cancer (AOC). GOG 218 and ICON 7 [13,14] phase III trials enrolled 1873 and 1528 women with newly diagnosed ovarian cancer, respectively. Most of them had advanced serous carcinoma but other histologies were included, such as clear-cell carcinoma and mucinous adenocarcinoma. The patients enrolled in the GOG 218 trial has incompletely resected stage III and stage IV diseases, while those included in ICON7 had stage I-IV diseases, thereby explaining their prognoses were different. Patients had been previously treated with primary debulking surgery (PDS) if feasible. Bevacizumab was combined to adjuvant carboplatin-paclitaxel regimen and then continued as a maintenance therapy every three weeks for 12 to 15 months. Both trials met their principal endpoints. Median PFS in GOG 218 was 14.1 months in bevacizumab-arm versus 10.3 months in the control arm, corresponding to a hazard ratio (HR) of 0.72 (95% confidence interval (CI) 0.63 to 0.82, *p* < 0.001). In ICON 7, median PFS was also improved from 17.4 months to 19.8 months, corresponding to a HR of 0.87 (95% CI 0.77 to 0.99, *p* = 0.04). However, OS was not significantly increased in the ITT analyses of both trials. An OS benefit of bevacizumab was found in a post-hoc subgroup analyses of patients at higher risk (Stage III with > 1 cm tumor residuals and stage IV) [15,16]. Adverse events related to bevacizumab included hypertension, hemorrhage, thromboembolic events and gastrointestinal perforations.

Interestingly, survival curves in both bevacizumab and non-bevacizumab arms tended to join rapidly after the discontinuation of bevacizumab thereby suggesting that the PFS benefit was observed only during bevacizumab maintenance treatment. The BOOST trial (ClinicalTrials.gov Identifier: NCT01462890) will determine if a prolonged administration of bevacizumab up to 30 months could further improve PFS. In both studies, PFS benefit associated with bevacizumab appeared to be limited to women with high-risk diseases, especially in those with residual macroscopic lesions after surgery or those with stage IV. Unfortunately, data are lacking about the potential interest of bevacizumab in patients with frontline CC-0 surgery, who were not included in the GOG 218 trial and represented a minority of patients in the ICON 7 trial. Patients with neoadjuvant chemotherapy (NACT) and IDS were not included in both trials. A recent ancillary study of the ICON7 trial suggested that bevacizumab improved OS only among patients with high-risk diseases and a chemoresistant disease (assessed by the modeled kinetic parameter KELIM) [17].

Although the exact role of bevacizumab in first line setting is still debated, its administration is recommended by international guidelines for patients with high-risk advanced ovarian cancer such as stage IV or suboptimally debulked disease [18,19].

## 3. DNA Repairs Defect and Poly (Adenosine Disphosphate-Ribose) Polymerase Inhibitors (PARPi)

### 3.1. DNA Damage Response and Repair Pathways Involved

**Figure 1:**
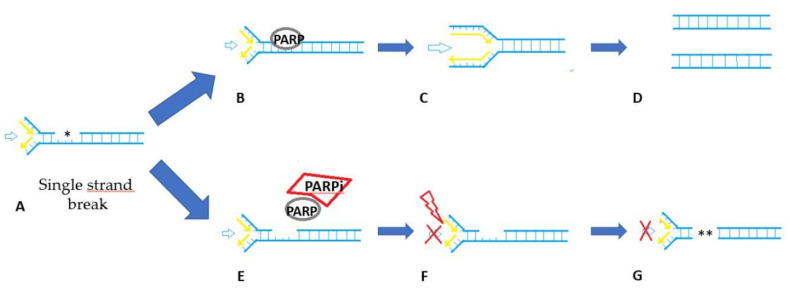
PARP inhibitor mechanisms. (**A**) DNA single strand break damage (SSB) appears and induce stress on the replication fork duplicating DNA in S phase. (**B**) Physiologically, the base excision repair (BER) pathway intervene through PARP enzyme. (**C**) SSB is fixe and replication proceed. (**D**) At the end of phase S the cell has 2 copies of DNA and mitosis continues. (**E**) However in the presence of PARP inhibitor (PARPi) the BER mechanism does not detect SSB and the PARP enzymes bind on the SSB are trapped. (**F**) The replication fork collapses in response to unrepaired SSB and PARP entrapment. (**G**) PARP trapping leads to double strand-break (DSB). In case of homologous repair-deficiency (HRd) phenotype, the DSB can be resolved by the non-homologous end-joining (NHEJ), which generates numerous mutations, resulting in genome instability and cell death.

Maintenance therapy with PARP inhibitors has initiated a new era in the management of high-grade serous ovarian cancer (HGSOC). PARP plays an essential role in the repair of single-strand DNA breaks, as a part of the base excision repair (BER). PARP inhibitors are trapped at the sites of single-strand breaks and the DNA repair process will require double-strand DNA breaks during the replication [20]. Usually, double-strand DNA breaks should be repaired by the protein complex leading to homologous recombination repair (HRR) activation, which includes BRCA1 and BRCA2 proteins, among others [21]. Tumor with HRR impairment or deficiency, such as *BRCA* mutated cancers, cannot repair double-strand DNA breaks induced by PARP inhibitors. The non-homologous end-joining (NHEJ) is a low fidelity repair pathway solicited to compensate the inoperative single-strand (BER) and double-strand repair mechanisms (HRR) which ultimately leads to the accumulation of genomic instability and cell death. This concept, known as synthetic lethality, has been the cornerstone of the therapeutic functioning of PARPi and is illustrated in Figure 1 [22]. HGSOC are known for their genome instability [23], a consequence of HRR deficiency found in about 50% of epithelial ovarian cancer, including those related to *BRCA1/2* mutations [24,25] or to mutations of other *HRR* genes such as *ATM*, *ATR*, *RAD 51*, *PALB2* [26,27]. As a consequence, if *BRCA* gene mutations were found to be associated with PARPi efficacy [28], other alterations of the *HRR* complex might also be predictive of PARPi activity and contribute to the PARPi-sensitive tumor phenotype beyond *BRCA* mutation, known as “BRCAness” [29]. In order to identify non-*BRCA* mutated patients likely to be sensitive to PARP inhibition, some companion tests have been developed. The most regularly used in clinical trials has been the myChoice test^®^ (Myriad Genetics, Salt Lake City, UT, USA), which combines three phenotypical markers (the so-called “genomic scars”) of HRD: loss of heterozygosity (LOH), telomeric allelic imbalance and large-scale state transitions in cancer cells [30]. The myChoice test^®^ (Myriad Genetics, Salt Lake City, UT, USA) relies on a quantitative score, that has been categorized using various thresholds for defining HRD positive (deficient) and HRD negative (proficient) patients.

### 3.2. From Pathophysiology to Recent Clinical Results

Historically BRCA 1 and 2 were the first described proteins harboring driver mutations in HGSOC. These germlines or somatic mutations are identified in 20–30% of HGSOC. SOLO1 [31] is a randomized, double-blind, phase III trial conducted in 391 patients with newly diagnosed, advanced high-grade serous or endometrioid ovarian cancers carrying BRCA 1 or 2 germline or somatic mutations. All patients had stage III or IV cancer and were in complete or partial response after platinum-based chemotherapy. Patients received olaparib (tablets—300 mg twice daily) or a placebo after chemotherapy. The maintenance treatment was continued for up to 2 years or until in the case of residual disease. The primary objective was PFS and the primary analysis of the trial was conducted after a median follow-up of 41 months. Median PFS from randomization, done at the end of chemotherapy, was 13.8 months in the placebo arm and was not reached in the olaparib arm (HR 0.30; 95% CI: 0.23 to 0.41, *p* < 0.01). The PFS rate at 3 years was 60% in the olaparib group against 27% in the placebo group. The median PFS to the first subsequent therapy were 51.8 months in the olaparib arm and 15.1 months in the placebo arm (HR 0.30; 95% CI: 0.22 to 0.40). Moreover, the times to second progression-free survival (PFS2) were not reached in the olaparib arm and 41.9 months in the placebo-arm, with an HR of 0.50 (95% CI: 0.35 to 0.72, *p* = 0.0002). At the time of PFS analysis, the interim OS data were immature. Those results led to Federal Drug Administration (FDA) and European Medicines Agency (EMA) approvals of olaparib as first-line maintenance therapy for women with *BRCA1/2*-mutated advanced stage, high-grade EOC.

Three pivotal phase III studies using different PARPi as maintenance treatments in first line setting were subsequently reported and have added substantial knowledge to the treatment of that disease.

#### 3.2.1. PARPi in Maintenance After Chemotherapy

PRIMA/ENGOT-OV26/GOG-3012 [32] was a prospective randomized phase III trial meant to assess the efficacy of the PARPi niraparib, after completion of fist line chemotherapy. Seven hundred thirty-three women were randomized. The trial included a population of patients with high risk diseases restricted to stage IV (30%) or stage III with residual tumor after primary debulking surgery (99% of operated patients), as well as patients (67%) treated with IDS after NACT. The trial was designed to assess the primary endpoint PFS with a hierarchical analysis in patients with HRD positive phenotype and then in the overall population. Patients were stratified on homologous recombination repair (HRR) status (HR deficient (HRd)) vs. HR proficient (HRp), the latter including unknown status). HRd population was defined as *BRCAm* status or a score > 42 using the myChoice test^®^ (Myriad Genetics, Salt Lake City, UT, USA), according to previous data coming from other trials. 373 patients (50.9%) were HRD positive, including 223 BRCA mutated patients. The progression-free survival was statistically longer in the HRd population (median PFS of 21.9 months versus 10.4 months) and in the overall population (median PFS of 13.8 months vs. 8.2 months) with an HR of respectively 0.43 (95% CI, 0.31 to 0.59, *p* < 0.001) and 0.62 (95% CI, 0.50 to 0.76, *p* < 0.001). OS data are not yet mature. The analysis of PFS in the HRp population was exploratory because this subgroup was not planned in the primary endpoint analysis. Moreover, conversely to the other trials and to the initial stratification scheme, the patients with unknown HR status were not associated with those with HRp tumors in the analysis. However, it indicated a modest benefit in favor of the niraparib arm over the placebo arm with a median PFS of 8.1 months vs. 5.4 months corresponding to an HR of 0.68 (CI 95%, 0.49 to 0.94). Most frequent and limiting AEs were thrombocytopenia (46% of patients, 28.7% of grade ≥ 3), anemia (63% of patients, 31% of grade ≥ 3) and nausea (57% of patients, 1.2% of grade ≥ 3). Analysis of health related quality-of-life scores reported no difference (Table 1).

VELIA/GOG-3005 [34] is a prospective randomized phase III trial aiming at assessing another PARPi, veliparib. 1140 patients stage III or IV diseases were enrolled. Unlike PRIMA [33] and PAOLA-1 [35], randomization was done before chemotherapy start. Patients received low-dose veliparib (150 mg twice daily) throughout chemotherapy followed by full dose (300 mg twice daily) in maintenance in the arm A; or veliparib only in combination with chemotherapy followed by placebo maintenance in the arm B; or placebo in combination with chemotherapy and during maintenance in the arm C. Patients were stratified according the germline BRCA mutational status. Patient HRR status was also assessed using the Mychoice test^®^ (Myriad Genetics, Salt Lake City, UT, USA) but with a lower threshold value at 33. Out of 1140 randomized patients, 298 (26%) had germinal or somatic *BRCA* mutations; 329 (29%) were HRd with *BRCAwt*; and 33% were HRp. Veliparib in the throughout arm A was associated with an improved PFS over chemotherapy alone but not in the concomitant veliparib arm B. The median PFS in the *BRCAm* cohort was 34.7 vs. 22 months with an HR of 0.44 (95% CI, 0.28 to 0.68, *p* < 0.001), in the HRd cohort it was 31.9 vs. 20.5 months corresponding to an HR of 0.57 (95% CI, 0.43 to 0.76, *p* < 0.001) and 23.5 vs. 17.3 months in the ITT cohort corresponding to an HR of 0.68 (95% CI, 0.56 to 0.83, *p* < 0.001). Chemotherapy dose-density was similar across all groups. Most frequent AEs in the throughout group were nausea (80% of patients, 8% of grade ≥ 3), anemia (64% of patients, 38% of grade ≥ 3) and thrombocytopenia (58% of patients, 28% of grade ≥ 3). One acute myeloid leukemia in the veliparib throughout arm A and one myelodysplastic syndrome was described in the veliparib combination arm B; and Nausea were responsible of 8% of treatment discontinuations. They were no clinically significant difference between the two arms in quality-of-life parameters (Table 1).

#### 3.2.2. PARPi with Bevacizumab in Maintenance Setting After Chemotherapy

PAOLA-1/ENGOT-ov25 [35] is a phase III randomized study meant to assess the benefit related to the addition of olaparib to bevacizumab in first-line maintenance therapy. The principal endpoint was PFS and the study was stratified on *BRCA* mutation status (germline or somatic *BRCAm* vs. wild type *BRCA*). After platinum-based chemotherapy, enrolled patients were randomized between bevacizumab maintenance (15 mg/kg every 3 weeks) in combination with olaparib (tablets: 300 mg bid/day) or with placebo. 806 patients were randomized. The randomization was stratified on the *BRCA* mutational status. Moreover, a subgroup analysis based on HRR status assessed using myChoice test^®^ (Myriad Genetics, Salt Lake City, UT, USA) categorized with a threshold at 42 was planned. 30% had stage IV HGSOC, 60% underwent complete cytoreduction, 70% had no evidence of disease or complete response to chemotherapy and 30% had a deleterious, germline or somatic BRCA mutation. The median PFS was statistically longer in the combination arm compared to the standard arm (22.1 months versus 16.6 months) corresponding to an HR of 0.59 (95% CI, 0.49 to 0.72, *p* < 0.001). The benefit was maintained in the *BRCAm* population. Median PFS was 37.2 vs. 21.7 months in favor of the combination corresponding to an HR of 0.31 (95% CI, 0.20 to 0.47) and in HRd e population (including *BRCAm* patients) with a median PFS of 37.2 vs. 17.7 months and an HR of 0.33 (95% CI, 0.25 to 0.45). In the HRp or unknown status subgroup, no benefit was seen in the olaparib arm. The most common adverse events in the combination arm were fatigue (53%, 5% of grade ≥ 3), nausea (53%, 2% of grade ≥ 3) and anemia (41% of patients, 23% of grade ≥ 3). The incidence of cardio-vascular or nephrotoxic adverse events frequently observed with bevacizumab was not higher in the combination arm. Myelodysplastic syndromes, acute myeloid leukemia and aplastic anemia were described in 6 patients in the olaparib arm (1% of the treated population). No differences in health-related quality of life were observed in the 2 arms (Table 1).

### 3.3. What Lessons Can We Learn and What New Questions are Being Raised?

The three trials highlight three biologically distinct populations of HGSOC: (1) those with germline or somatic *BRCA* mutations carriers; (2) those with HRd phenotype (with or without *BRCA* mutation); (3) and those with HRp phenotype.

PARPi maintenance was undoubtedly associated with higher PFS in HRd populations (with or without *BRCA* mutation). However, the PFS improvement related to PARPi in HRp patients remains unclear. Thus, determining the HRR status before starting maintenance treatment after chemotherapy is of high interest. However, the recent niraparib approval by FDA was not conditioned by the necessity of having the HRR status, whilst those of olaparib + bevacizumab requires a HRD positive status (*BRCA* mutation or genomic instability defined using a FDA approved assay) [36,37].

We are still waiting for OS data, which will be available in the coming years. However, the PFS 2 (e.g., PFS assessed with the treatment following progression under or after first-line olaparib) reported in the SOLO-1 trial was clearly encouraging, in favor of the olaparib arm [31].

#### 3.3.1. PARPi in Front-Line or Later?

In the absence of any demonstration of an OS benefit favoring the introduction of a PARPi in maintenance after front-line chemotherapy, no definitive recommendation can be made on the best therapeutic sequence. It is still uncertain whether PARPi maintenance treatment in a first line setting will be associated with overall survival gain, as has been demonstrated in platinum-sensitive relapse among *BRCA* carriers.

An OS benefit with olaparib maintenance treatment was observed in SOLO-2 trial [38]. We still do not know if such a similar gain could later be reported in PRIMA trial. However, a benefit in PFS was reported in a recent meta-analysis for patients of all molecular subgroups in platinum-sensitive recurrent ovarian cancer (ROC) [39]. Because of the range of PFS gain observed in first-line, the introduction of PARPi at first-line could limit the number of patients likely to die at first tumor progression, along with assumed to experience platinum-resistant relapse within 6 months after the end of platinum based-chemotherapy and who would not benefit from PARPi during their recurrence.

The current approvals of PARP inhibitors in patients with recurrent diseases exclude those who were previously treated with PARPi, whether they have progressed during maintenance on PARPi or if they relapsed after the end of the maintenance. It is unclear whether the mechanisms of biological resistance are similar in the two cases and therefore whether there is an interest in re-challenging these patients pre-treated with PARPi.

In the meantime, it is rational to consider that PARPi should be administered to the maximum number of patients, either in fist line or in platinum-sensitive relapse, given the amplitude of the PFS benefit.

#### 3.3.2. PARPi in Combination with Chemotherapy?

No difference in the likelihood of complete interval debulking surgery was found in between the experimental arm and the veliparib arms, which suggests that veliparib did not increase the chemotherapy efficacy. However, most of enrolled patients were treated primary after debulking surgery, thereby reducing the power of the analyses regarding this hypothesis. Moreover, the VELIA trial, did not integrate a “maintenance-only arm” with veliparib started after chemotherapy completion, as assessed in PRIMA and PAOLA trials. As a consequence, it is difficult to discriminate the effect of veliparib given concurrently with chemotherapy, with those of the maintenance. The CT dose-intensity was equal with and without veliparib while the grade 3 frequencies of hematologic AEs were comparable in both combination and throughout arms. For instance, grade 3 anemia was 26% in control arm, 41% in combination and 39% in throughout arms. Grade 3 neutropenia were 49% in control arm, 62% in combination and 58% in throughout groups, respectively. However dose interruption, reduction and discontinuation were comparable with PAOLA-1 results.

Pre-clinical data suggest that PARPi could have a neuroprotective effect against platin-induced distal neuropathy [40]. In VELIA, PAOLA-1 and PRIMA no significant decrease in neuropathy were noticed in the experimental arms. However, few details were available on how the occurrence of neuropathy and its intensity were collected, thereby making the interpretation complicated. If this therapeutic effect, was confirmed, it would favor the use of PARPi in combination with CT.

#### 3.3.3. PARPi in Combination with Bevacizumab?

The actual benefit offered by the addition of bevacizumab to a PARPi, compared to a PARPi alone, remains unclear, since the PAOLA-1 trial did not integrate a olaparib-alone arm.

In the *BRCAm* subgroup the benefit of adding bevacizumab to PARPi is uncertain. The relative benefit reported with olaparib + bevacizumab combination compared to bevacizumab alone in PAOLA-1 trial was similar to those observed with olaparib compared to placebo in SOLO-1 trial, thereby confirming the efficacy of olaparib in patients with *BRCA* mutated tumors. In vitro studies suggested a synergistic effect with the association of PARPi with anti-angiogenic properties. Indeed, hypoxia may increase DNA instability requiring DNA repair system, including homologous recombination. The favorable data of the combination niraparib + bevacizumab in AVANOVA trial [41] support this hypothesis. In the phase II trial AVANOVA, 97 women with platinum-sensitive recurrent ovarian cancer were randomized to receive niraparib alone or in combination with bevacizumab. PFS was increased in the combination arm in the overall ITT population as well as in HRd and HRp subgroups. Like in PAOLA-1 trial, the toxicity profile of PARPi and bevacizumab appeared acceptable while patient-reported outcomes revealed no relevant effect of treatment on quality of life. These results would support the superiority of the combination over PARPi in monotherapy. However, when compared to those observed in SOLO-1 trial, the long median PFS observed in the experimental arm of PAOLA-1 trial are more consistent with additive effect of bevacizumab and olaparib than a synergistic effect [42].

Even if cross-trial comparisons have major limitations, it can be noted that patients in the control group of the PAOLA-1 trial had substantially longer PFS, as compared with patients in the control group of the PRIMA trial. On the other hand, the PAOLA-1 trial population was of better prognosis. A population adjusted indirect comparison of high-risk patient included in PAOLA-1 and PRIMA suggested an improved efficacy of olaparib and bevacizumab over a treatment with niraparib or bevacizumab alone [43]. If we assume that olaparib and niraparib have the same efficacy profile (as observed in recurrent setting), the PFS difference in PAOLA-1 and PRIMA trials could only be explained by the role of bevacizumab.

It is likely that PARPi and bevacizumab may deal with different aspects of ovarian cancer diseases. PARPi seem to be more effective in patients with minimum residual lesions and highly platinum-sensitive diseases [33,34,35,44], whilst bevacizumab is likely to be more beneficial in patients with bulky lesions [15,45] and chemoresistant diseases [17]. As a consequence, it would be rationale to favor the co-prescription of olaparib and bevacizumab in the patients for whom bevacizumab was regarded as a standard according to GOG 218 and ICON 7 trials (e.g., “high-risk” patients, with residual disease and stage IV). Further trials will be necessary to address that specific question.

It should be noted that the combination of bevacizumab and olaparib was associated with the highest discontinuation rate (20%) of maintenance treatment among the 3 studies. However, the presentations of the data differed between the trials. In PAOLA 1, the reported treatment discontinuations were those related to adverse events and patients’/investigators’ decision, whilst they were related to toxicity only in PRIMA trial.

#### 3.3.4. What is the Benefit of PARP Inhibitors in the HRp Population?

In the PRIMA study, the HRR status was a stratification criteria. A benefit with niraparib was reported in patients with HRd tumors, one the 2 primary endpoints but also in those with HRp tumors which was not included in the primary objective design. Moreover contrarily to the methodology used for PAOLA-1 and VELIA trials, the patients with unknown HRR status were not included in this subgroup. Subsequent analyses using more standard log-rank tests confirmed the prognostic value of niraparib in the subpopulation of patients with HRp tumors [46].

In the PAOLA-1 trial, no benefit was seen with the combination olaparib + bevacizumab in the subpopulation of patients with HRd or HRR unknown tumors.

As the analyses of the HRp subgroup of the PRIMA trial are exploratory and as no comparative data are available, no definitive conclusions can be drawn on a differential effect of niraparib, olaparib and veliparib on this subgroup.

#### 3.3.5. What are the Clinical Differences Across PARPi: What Specific Adverse Events to Expect?

Due to the lack of OS benefit and very similar PFS, AEs represent the most prominent clinical and significant distinction among PARPi, especially regarding the AEs ≥ grade 3. Although there are no significant changes in the quality of life, they must be tracked down as a way of limiting the risk of maintenance therapy discontinuation. Many AEs are class effects, such as cytopenia, but some are more dependent on the drug involved. A meta-analysis detected no significant differences in all grade hematologic AEs, more frequent in the initial months, however severe hematologic AEs were found to be more frequent with niraparib [47]. Niraparib is now recommended to be started at 200mg daily in patients with weight < 77 kg or with a low platelet count < 150 × 109 cells/L after a retrospective analysis of the NOVA trial [48]. The PRIMA trial was amended accordingly in 2017. Moreover hypertension were reported in 19% of patients enrolled in the NOVA trial.

Rucaparib demonstrated efficacy in monotherapy in heavily pretreated AOC [49,50], in *BRCA* carriers or HRd population and in maintenance therapy in ROC [51,52,53]. In the trials, rucaparib-treated patients reported intense abdominal pain [47], associated with grade 3–4 transient transaminase elevation in 10% of cases [52]. It was developed early, without association with bilirubin increase and tended to normalization after few weeks. Serum creatinine level increases were also reported in 20% of patients with rucaparib.

The other side effects such as fatigue, diarrhea, dermatological toxicities were generally grade 1–2 and were reported in similar manners among patients treated with olaparib, niraparib, veliparib and rucaparib. Myelodysplastic syndrome and acute myeloid leukemia are severe class-related AEs found in 1% of patients and must be considered in the case of prolonged pancytopenia.

#### 3.3.6. What Perspectives for the Companions Diagnostic Tests Used?

The methodology to be used for assessing the HRR status is another issue. Indirect exploration through evaluation of loss of heterozygosity (LOH) is a promising path [54]. Results of ARIEL 2, a trial evaluating rucaparib (PARPi) in a majority of wild-type *BRCA* (*BRCAwt*) platinum-sensitive recurrent patients, defined two subgroups of patients without *BRCA* mutation, either as “BRCA-like” or “biomarker negative,” depending on the high rate of LOH. The “BRCA-like” subgroup responded better to PARPi [50]. According to these encouraging results, ARIEL 3 prospectively evaluated a HRd test based on LOH in platinum-sensitive recurrent patients [52]. This test was developed in collaboration with Foundation Medicine [55]. Median PFS were higher in patients with carcinomas characterized by high LOH compared with those with low-LOH (median PFS 9.7 months vs. 6.7 months). If LOH helped discriminate those who benefited from rucaparib or not, it was not fully predictive either: 30% of patients BRCAwt and low-LOH experienced long PFS > 1 year compared with 5% in the placebo group. As a consequence, LOH cannot be considered as an ideal companion test.

MyChoice test^®^, elaborated by Myriad Genetics (Salt Lake City, UT, USA), combines three assays in a score to assess HRR status: LOH, large-scale state transitions in cancer cells and telomeric imbalance.

A positive HRR status using MyChoice test^®^ (Myriad Genetics, Salt Lake City, UT, USA), means that the tumor is characterized by mutations in *BRCA1* and *BRCA2* genes or by a high Genomic Instability Score (GIS) defined using genome-wide single nucleotide variants in DNA extracted. It classifies patients into HRR status subgroups (such as HRd or HRp, *BRCA* mutation status positive or negative) but also provide data on deleterious mutations or suspect genetic variant if the detected mutation is unknow.

The initial cutoff to categorize tumor phenotype as HRd or HRp was initially set at 42 (100 corresponding to a typical HRd profile). It was supported by the data that led to the approval of niraparib for HRd patients [32,56]. The same cutoff was chosen for PRIMA and PAOLA-1 trials, whilst VELIA selected a lower threshold at 33 [57,58]. This choice mathematically led to a higher number of patients in the HRd subgroup and might have impacted PFS outcomes among both HRd and HRp subgroups.

FDA granted approval to this test centralized in the US, with an approximative cost of 4000 USD per test. This price added to the cost of the PARPi maintenance treatment raises the question of the cost-effectiveness of the comprehensive treatment sequence strategy based on HRd testing. This perspective should be considered more broadly than thus through the ovarian cancer window. Indeed PARP inhibitor maintenance is being investigated in patients with HRd tumors of other origins such as breast, pancreas and prostate cancers [59,60]. Developing an institutional companion test suitable for these different oncologic spheres would be of great interest in view of the promising utility of HRd testing.

To make things even more complicated, HR is actually as a system composed of many genes. There are now many in vitro data on the functions of them. Outside *BRCA1/2*, the predictive values of alterations (mutation, deletion) on these genes considered individually are still unknown. Many projects are trying to develop panels of HR genes but their validation will take time, thereby explaining the relevance of above approach based on the “genomic scar”.

The administration of a PARPi for maintenance after front-line chemotherapy would increase the number of patient candidates for these drugs, as patients would no longer be selected based on platinum-sensitivity. The use of platinum-sensitivity as a biomarker to define which patient will benefit of PARPi is based on differential efficacy observed in non-comparative trials but a significant efficacy has been reported with PARPi for the treatment of BRCA carriers with a platinum resistant disease [50,61].

The early introduction of PARPi in the strategy, based on a biological selection of patients of patients likely to benefit from the treatment rather than on the platinum sensitivity, might improve long-term outcomes. The long-term outcomes of the PRIMA and VELIA trials will give critical information on this pending question.

## 4. Conclusions

The success of the first line treatment in ovarian cancer patients, will rely on the combination of 2 main medical-surgical parameters: (1) the likelihood of the complete primary or interval debulking surgery and (2) the efficacy of systemic medical treatment based on chemotherapy along with targeted agents involved on the angiogenesis and on DNA repair. Interestingly, if these 2 parameters are complementary, they are also deeply entangled, in the sense that the likelihood of complete surgery is highly related to the chemosensitivity [52]. Moreover the efficacy of PARPi is also dependent on the platinum-sensitivity, thus confirming the major of the primary tumor chemosensitivity [52].

If the impact of the completeness of debulking surgery is well recognized, the primary chemosensitivity has still been poorly integrated in the comprehensive management of ovarian cancer. This aspect is, however, major, since a growing bulk of data suggest that the prognosis of patients with highly chemosensitive disease will undoubtedly improve in the next year with the broader prescription of PARPi since these patients will have full benefit from PARPi maintenance, in addition to their higher probability of complete surgery.

The best characterization of this chemosensitivity through modeled CA125 kinetics with KELIM or through somatic HR status with genomic scarring or gene panel will have to be improved. In fact, the definition of the HRR phenotype faces several issues. If the companion test validated in the phase III trials makes it possible to guide the treatment decision, the threshold of positivity remains unclear. Moreover, the cost of these explorations may be a limit in the years to come, especially as the search for the HRR status seems to be an issue in several relatively common solid tumors. Furthermore, the understanding of the different genes involved in the HRR pathway remains limited and individual consideration of these mutations in future prospective trials would provide important answers.

These data delineate a population of patients with poor prognosis, that would warrant further innovative approaches. Immune checkpoint inhibitor (ICI) such as anti-programmed-death ligand-1 (PD-L1) are being evaluated in front-line and maintenance therapy, in combination with chemotherapy, bevacizumab and PARPi. Four clinical trials are still recruiting patients: ATHENA trial (ClinicalTrials.gov Identifier: NCT03522246 ATHENA) [62], DUO-O trial (ClinicalTrials.gov Identifier: NCT03737643 DUO-O) [63], from AGO group, ENGOT-ov43 (ClinicalTrials.gov Identifier: NCT03740165 ENGOT-ov43) [64], from BGOG and FIRST trial (ClinicalTrials.gov Identifier: NCT03602859 FIRST) [65], from GINECO group (Table 2). PARP inhibition might increase cancer antigens, the number of DNA mutations and the production of interferon type I, therefore enhance the immune response against cancer cells [66]. The JAVELIN OVARIAN trials failed to demonstrated any benefit with avelumab alone, an anti PD-L1 agent, in combination with CT [67,68,69]. In JAVELIN OVARIAN 100 trial, patients were randomized to receive avelumab in maintenance in first-line setting in combination or following chemotherapy. No superiority in OS or PFS were found with avelumab. No patient subgroup according to PD-L1 or BRCA biomarkers benefited clearly of ICI [68]. In platinum-resistant disease, combination of avelumab and pegylated liposomal doxorubicin failed to improve PFS and OS in comparison with CT or avelumab alone [69].

On the other hand, the addition of anti-VEGF agents increase lymphocyte T infiltration in the tumor microenvironment and might add a synergistic effect with ICI.

The combination of PARPi with others anti-angiogenic agent without CT might be an alternative to CT. A randomized phase II trial compared the efficacy of cediranib, a tyrosine kinase inhibitor which selectively inhibits all three vascular endothelial growth factor receptors (VEGFR-1, -2 and -3), combined to olaparib or olaparib monotherapy, in platinum-sensitive ROC [70]. Patients were naïve of PARPi and bevacizumab. The combination prolonged PFS compared to olaparib in the intent to treat population. A differential benefit of the combination was observed in the BRCAwt population Despite promising results, the phase III failed to prove a benefit in PFS or OS for the combination in comparison with olaparib alone or standard CT [71]. Nevertheless, the perspective of a therapeutic-line free of CT with more favorable toxicity profile seems to be achievable. Ongoing phase III studies of cediranib and olaparib combination will add data in maintenance setting after CT in platinum-sensitive ROC (ClinicalTrials.gov Identifier: NCT03278717) and platinum-resistant ROC versus CT (ClinicalTrials.gov Identifier: NCT02502266).

Tumor progression after treatment with PARPi means the development of one or more resistance mechanisms. Primary resistance occurs in tumors with HRp phenotype due to the preservation of the HRR function. In HRd, further alterations in the repair pathways of DNA, acquired under PARPi treatment, may explain secondary resistance to these PARPi. One mechanism is the reactivation of the HR pathway through the restoration of BRCA protein. This can be achieved through secondary mutation in *BRCA* gene restring the open reading frame (*BRCA* reversion), the loss of *BRCA* promoter methylation, a *BRCA1* C-terminal domain mutation that upregulated BRCA1 protein or initiation of HR mechanism independently of *BRCA* [23,72,73]. The reactivation of the HR pathway could also be due to the decreased activity of the NHEJ pathway which is initially overactivated in case of HRd phenotype treated with PARPi, trough downregulation of the promoter protein 53BP1 [74]. Reduced cellular availability of PARPi through overexpression of the gene that encode the MDR1 efflux protein could also be a resistance mechanism with PARPi [75,76]. Many trials assessing PARPi activity have excluded patients with prior PARPi exposure. As a consequence, we lack clinical evidence of PARPi efficacy after re-exposure, either with the same molecule or with a different one associated with a distinct resistance profile. The results of the OREO study (ClinicalTrials.gov Identifier: NCT03106987), which evaluates the relapse of PARPi in women already treated on previous maintenance, are awaited. Inhibitors of the cell cycle checkpoints such as ATR, WEE1 and CHK1 are being evaluated in combination with PARPi. The DUETTE trial (ClinicalTrials.gov Identifier: NCT04239014) is assessing the combination of ceralasertib (ATR inhibitor) and Olaparib in platinum-sensitive ovarian cancer previously treated with PARPi. CHK1/2 inhibitor demonstrated in phase II trial an ORR of 29% in non-*BRCAm* carriers [77]. Pre-clinical data suggest that WEE1 inhibitors could potentiated the PARP trapping effect of PARPi [78]. Other inhibitors are currently tested in combination with PARPi (ClinicalTrials.gov Identifier: NCT03057145). Epigenetic resensitization through DNA methyltransferase (DNMT) inhibitors could be a synergistic pathway in combination with PARPi since DNMT treatment may induce the BRCAness phenotype [79,80].

In order to increase the rate of prolonged remission in patients with AEOC, it seems necessary to optimize the maintenance treatment, in addition to optimal surgery if it is achievable. Several recent trials support the utilization of PARPi in patients with HRd status, representing 50% of them. In phase III trials, PARPi significantly prolonged PFS in BRCA mutated patients but also in patients with HRd phenotype. In Table 3 we propose treatment options for AOC.

## Figures and Tables

**Table 1:** Phase III trials of first-line PARP inhibitors.

Trial	Patients (*n*) *BRCAm* (%) HRD + (%)	Design	Median PFS (Primary End Point)	Any Grade > 3 AEs (%)	AEs Leading to Dose Interruption (%), Reduction (%), Discontinuation (%) in Experimental Arms
PRIMA [33]	733 *BRCAm* 30 HRd 51	Niraparib vs. placebo maintenance	HRD +: HR 0.43; 95% CI 0.31–0.59; *p* < 0.001 ITT: HR 0.62; 95%CI 5–76; *p* < 0.001	71 vs. 19	80 71 12
VELIA [34]	1140 *BRCAm* 26 HRd 55	Veliparib with CT only, veliparib with CT and maintenance, CT only and placebo	*BRCAm*: HR 0.44; 95%CI 28–68; *p* < 0.001 HRD +: HR 0.57; 95%CI 43–76; *p* < 0.001I TT: HR 0.68; 95%CI 56–83; *p* < 0.001	88 vs. 77	Combination: 58, 6, 11 Maintenance: 41, 24, 19
PAOLA-1 [35]	806 *BRCAm* 29 HRd 48	Olaparib and bevacizumab vs. placebo and bevacizumab	ITT: HR 0.59; 95%CI 49–72; *p* < 0.0001	57 vs. 51	54 41 20

*BRCAm* = *BRCA* mutation; HRD + = Homologous recombination deficiency positive; PFS = progression free survival; AE = adverse events; HR = hazard ratio; CT = chemotherapy.

**Table 2:** Phase III frontline trials associating Immune Checkpoint inhibitor (ICI) and PARP inhibitors ± bevacizumab.

Trial	Patients (*n*)	PARPi	ICI	CT	Maintenance	Completion
ATHENA [62]	1000 (4 : 4 : 1 : 1)	rucaparib	nivolumab	Per SOC	Rucaparib + nivo Rucaparib + plb Nivolumab + plb Plb + plb	December 2024
FIRST [65]	900 (2 : 1 : 1)	niraparib	TSR-042	CP + TSR-042 CP CP	Niraparib + TSR042 Niraparib plb	June 2020
ENGOT-Ov43 [64]	1500 (1 : 1 : 1)	olaparib	pembrolizumab	CP + pembro CP + pembro CP	Pembro + Olaparib Pembro + plb Plb + plb	August 2025
DUO-O [63]	927	olaparib	durvalumab	CP + BEV + D CP + BEV + D CP + BEV	BEV + D + Olaparib BEV + D BEV	May 2022
JAVELIN [67]	720 (1 : 1 : 1)	talazoparib	avelumab	CP + A CP CP + BEV	Avelumab + talazoparib Talazoparib BEV	Closed/ futility

Phase III frontline trials associating Immune Checkpoint inhibitor (ICI) and PARP inhibitors ± bevacizumab. Plb = placebo, SOC = standard of care, CT = chemotherapy, CP = carboplatin-paclitaxel, pembro = pembrolizumab, D = durvalumab, BEV = bevacizumab, A = avelumab.

**Table 3:** Proposition of maintenance setting after standard of care chemotherapy in front-line in advanced serous ovarian cancer.

Clinical Presentation	HRd	HRp
low risk	PARPi	No PARPi No bevacizumab
high risk (CC-1,2,3 or stage IV or ascites/pleuritis)	PARPi and bevacizumab	bevacizumab

HRd = Homologous recombination deficient; HRp = Homologous recombination proficient; CC= completeness of cytoreduction.
